# Supplementary material for: Evidence supported by Mendelian randomization: impact on inflammatory factors in knee osteoarthritis
Source: Front Med (Lausanne). 2024 May 28;11:1382836. doi: 10.3389/fmed.2024.1382836 (PMC11165061; doi:10.3389/fmed.2024.1382836)
Supplement: Supplementary file 1 [file Data_Sheet_1.ZIP › data/GCST90274797_5e6/797.leaveoneout.pdf]

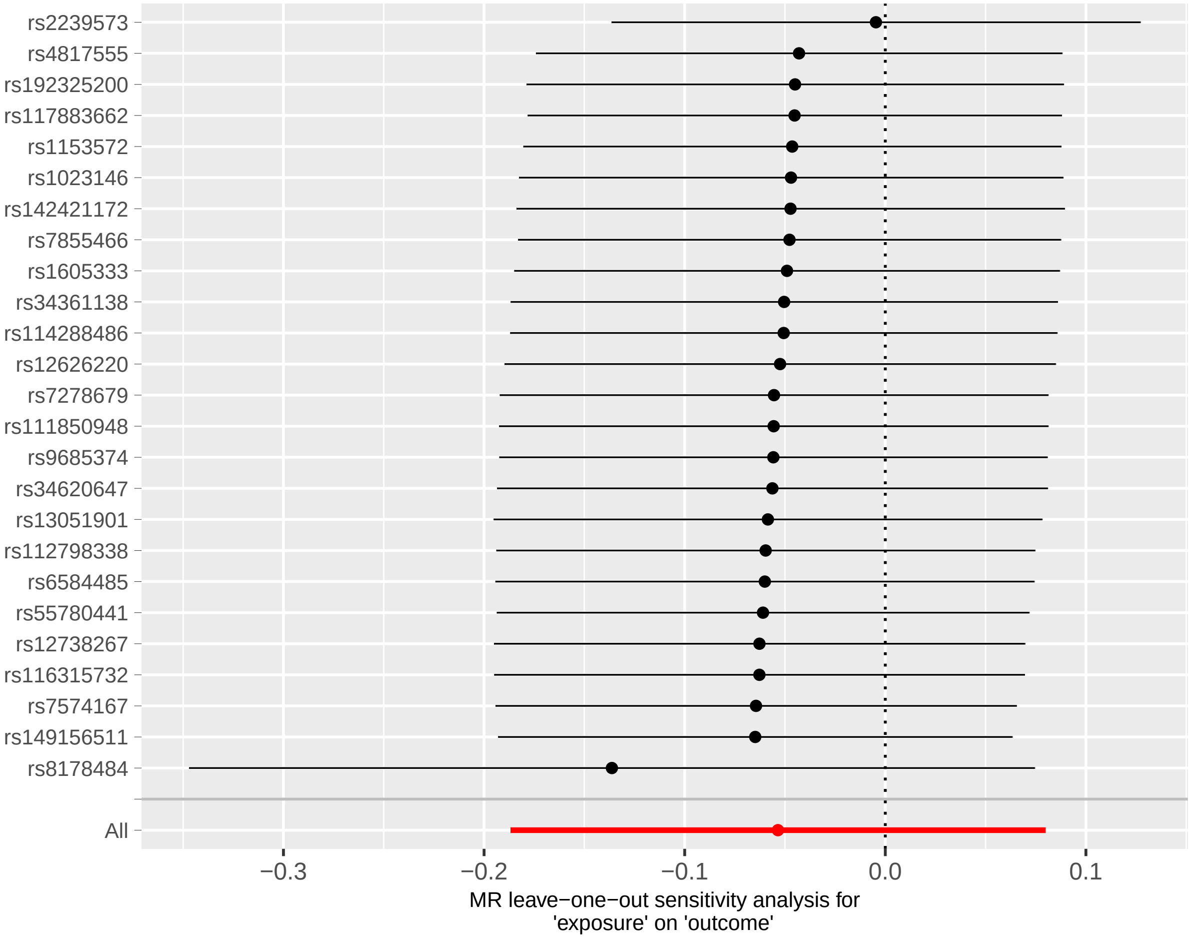
rs2239573
rs4817555
rs192325200
rs117883662
rs1153572
rs1023146
rs142421172
rs7855466
rs1605333
rs34361138
rs114288486
rs12626220
rs7278679
rs111850948
rs9685374
rs34620647
rs13051901
rs112798338
rs6584485
rs55780441
rs12738267
rs116315732
rs7574167
rs149156511
rs8178484
All
−0.3
−0.2
−0.1
0.0
0.1
MR leave−one−out sensitivity analysis for
'exposure' on 'outcome'
